# DNA Methylation and Chromatin Remodeling: The Blueprint of Cancer Epigenetics

**DOI:** 10.1155/2016/6072357

**Published:** 2016-03-28

**Authors:** Dipanjan Bhattacharjee, Smita Shenoy, Kurady Laxminarayana Bairy

**Affiliations:** Department of Pharmacology, Kasturba Medical College, Manipal University, Manipal, Karnataka 576104, India

## Abstract

Epigenetics deals with the interactions between genes and the immediate cellular environment. These interactions go a long way in shaping up each and every person's individuality. Further, reversibility of epigenetic interactions may offer a dynamic control over the expression of various critical genes. Thus, tweaking the epigenetic machinery may help cause or cure diseases, especially cancer. Therefore, cancer epigenetics, especially at a molecular level, needs to be scrutinised closely, as it could potentially serve as the future pharmaceutical goldmine against neoplastic diseases. However, in view of its rapidly enlarging scope of application, it has become difficult to keep abreast of scientific information coming out of various epigenetic studies directed against cancer. Using this review, we have attempted to shed light on two of the most important mechanisms implicated in cancer, that is, DNA (deoxyribonucleic acid) methylation and histone modifications, and their place in cancer pathogenesis. Further, we have attempted to take stock of the new epigenetic drugs that have emerged onto the market as well as those in the pipeline that offer hope in mankind's fight against cancer.

## 1. Introduction

Tumorigenesis, the process of development of cancerous cells, till recent times, was believed to be a by-product of aberrant genetic mutations alone. However, recent evidences have pointed to an important role of epigenetics in the development of cancer. The term “epigenetics,” a portmanteau of “epigenesis” and “genetics,” can be summed up in the following few words: “*Epigenetics refers to both heritable changes in gene activity and expression (in the progeny of cells or of individuals) and also stable*,* long-term alterations in the transcriptional potential of a cell that are not necessarily heritable*” [1]. The field of epigenetics revolves around the concept of “epigenetic landscape” at its core. In a very rudimentary form, epigenetic landscape has been designated to represent the intra- and extracellular environment and epigenetics refers to the study of the various interactions between the intracellular and extracellular environment that in turn decide the cell's fate. It has been hypothesised that the “epigenomic landscape,” governing the cellular expression, becomes extensively warped in cancerous cells [2]. This distortion is due to the deviations from the regular DNA methylation and histone modification patterns seen in normal cells. These alterations in the epigenetic regulatory machinery can bring about dysregulation in genetic expression patterns either by the activation of the oncogenes [3] or by the silencing of tumor suppressor genes. Thus, epigenetic dysregulation can lead to development and perpetuation of cancerous states. However, epigenetic regulatory machinery, being reversible and dynamic in nature, is shorn of the rigidity associated with genetics and possesses the potential to either cause or cure diseases, especially cancer. Hence, epigenetics, especially at a molecular level, needs to be examined closely, as it could serve as the future pharmaceutical goldmine. In light of this, it is our endeavour here to shed light on the implications of the two most prominent epigenetic mechanisms, that is, DNA methylation and histone modification, in the pathophysiology of cancer. Further, we attempt to take stock of the new epigenetic drugs that have emerged onto the market as well as those in the pipeline that offer hope in mankind's fight against cancer.

## 2. Cancer and DNA Methylation

DNA methylation primarily involves addition of methyl groups to the 5′ carbon at cytosine residues preceding guanine nucleotides, linked together by phosphate bonds (CpG) utilizing a methyl donor like S-adenosylmethionine. The CpG rich foci are asymmetrically arranged throughout the genome, clustered primarily in short CpG rich DNA sequences termed as “CpG islands” and regions of large repetitive sequences like centromeric repeats, retrotransposon elements, and so forth [4, 5]. DNA methylation, brought about by a class of enzymes termed as DNA methyl transferases (DNMT), target these CpG islands. There exist four DNMTs, that is, DNMT 1, 2, and 3a and 3b [6] of which DNMT 1 and DNMT 3 have been observed to play a pivotal role in DNA methylation.

DNA methylation can deter the process of transcription by inhibiting the binding of the transcriptional factors with the target sites as seen in case of* c-myc* and many other genes [7]. On the other hand, the methylated cytosine residues act as the site for docking of various methylated DNA binding proteins (MBD1, MBD2, MBD3, and Mecp2) that are recognized by various histone modifying enzymes like histone deacetylases (HDACs), which in turn can bring about gene repression [8–10].

A normal cell is characterized by genome wide methylation with the exception of CpG (cytosine-phosphate-guanine) islands, which are unmethylated [11]. However, by virtue of various triggers in cancerous cells, whose origins are yet to be understood properly, certain events are set in motion which lead to the hypomethylation of entire genome with the exception of CpG island promoters, which undergo hypermethylation [12].

### 2.1. Role of Hypomethylation in Cancer

Extensive DNA hypomethylation is essential for tumorigenesis as its occurrence at repetitive elements can produce an increase in genomic instability by advocating chromosomal rearrangements [12, 13]. Further, retrotransposons' hypomethylation leads to their activation. This in turn can lead to the translocation of retrotransposons to other genomic regions, which in turn could propagate the genomic instability [14]. Some of the best examples to exemplify the role of DNA hypomethylation in development of cancer have been illustrated here. DNA hypomethylation has been implicated in the activation of the growth promoting genes such as related-RAS (R-Ras) and mammary serine protease inhibitor (MAPSIN) for gastric carcinoma, S-100 in case of colonic cancer, melanoma-associated antigen (MAGE) in melanoma [15], and loss of imprinting, especially of insulin like growth factor 2 (IGF-2) as seen in Wilms' tumor [16] and colorectal cancer [17].

### 2.2. Role of Hypermethylation in Cancer

Contrastingly, CpG island hypermethylation can induce tumorigenesis by shutting down the expression of tumor suppressor genes. This can be achieved by direct action over tumor suppressor genes as well as indirectly by silencing of the concerned tumor suppressor genes' transcription factors and preventing the expression of DNA repair genes. Hypermethylation of Rb promoter gene (retinoblastoma associated tumor suppressor gene) was among the earliest instances to have been unearthed, where hypermethylation of the CpG promoter island site led to the silencing of the tumor suppressor gene and subsequently promotion of retinoblastoma malignancy [18]. A few other such tumor suppressor genes that undergo silencing due to hypermethylation include p16 and BRCA1 [19]. These genes are crucial to processes like cellular adhesion, apoptosis, and angiogenesis that are involved in the development and progression of cancer. On the other hand, hypermethylation of CpG promoter regions induced silencing of transcription factors as seen with RUNX3, GATA-4, and GATA-5 in esophageal, colorectal, and gastric cancers, respectively, leads to downstream target inactivation of the tumor suppressor genes, which in turn can lead to propagation of cancer cells [20, 21]. Further, DNA repair genes like MLH1 and BRCA1 on being silenced allow the accrual of many other genetic lesions, which in turn accelerates the progression of cancer.

In the light of growing evidence that progressively bolstered the claim that DNA hypermethylation plays a critical role in tumorigenesis, questions then arose as to how can selective targeting of genes by the DNA methylation machinery be carried out? One line of thinking is that CpG island specific methylation may be guided by a nucleotide sequence specific mechanism that in turn directs the DNMTs to their respective genes based upon their association with the oncogenic transcription factors. PML-RAR fusion protein guided abnormal hypermethylation and specific target promoter genes' silencing as seen in acute promyelocytic leukemia are a classic example to illustrate this [22]. Further, as has been observed in various types of cancer, large stretches of DNA tend to undergo methylation. This may lead to hypermethylation of the CpG islands by virtue of them being located within genomic regions that may have undergone large scale epigenetic reprogramming [23]. Apart from these two possible mechanisms, it has also been hypothesised that histone marks may also play a significant role in CpG island specific* de novo* DNA hypermethylation. Further light has been shed over this mechanism in the subsequent sections.

### 2.3. TET Proteins: The Master Key of DNA Methylation

Despite hypomethylation and hypermethylation producing diametrically opposite results in cancerous tissues, both these mechanisms tend to coexist in the same tumor, though afflicting different regions within the genome. Both these mechanisms can interact in many manners and at various levels to produce multiple subphenotypes of cancer. Further, the reversibility of DNA methylation epigenetic process adds to the complexity of the cancerous tissue's epigenome as it renders the epigenetic regulatory processes open to modification by various cellular environment changes. This again goes against the belief that DNA methylation could be a stable form of chromatin modification. High resolution pan-genome DNA methylation mapping in pluripotent and differentiated cells has further underlined the flexible nature of DNA methylation. This could be attributed to the presence of an enzymatic system that may be responsible for either completely abolishing or maybe altering this epigenetic modification [24]. This hypothesis, attempting to explain the dynamic nature of DNA methylation, received a shot in the arm with the discovery of ten-eleven translocation (TET1–3) group of proteins. TET group of proteins is etymologically associated with a recurrent chromosomal translocation t(10; 11)(q22; q23) that apposes mixed-lineage leukemia or myeloid-lymphoid leukemia (MLL) gene with TET1 protein in a few acute myelocytic leukemia (AML) patients. This family of proteins functions as mammalian DNA hydroxylase that converts 5-methyl cytosine (5 mC) to 5-hydroxymethylcytosine (5 hmC), which, on further oxidation, ends up yielding various oxidation products, like 5-formylcytosine (5 fC) and 5-carboxylcytosine (5 caC). Though further studies are needed to fully establish the biological role of these oxidation derivatives, it is believed that these derivatives could be the critical intermediates in DNA methylation, be it active or passive. Further, they could also play an important role in either preventing or even enhancing the attachment of methyl CpG binding domain (MBD) proteins and could also regulate the recruitment of chromatin regulators. Additionally, the genome wide distribution of 5 hmC gives rise to the notion that 5 hmC and TET proteins in all probability may influence both transcriptional activation and silencing [25].

### 2.4. DNA Methylation and Its Therapeutic Applications

In light of the dynamic and far reaching effects of aberrant DNA methylation, it is not surprising to note that hypomethylating agents (Table 1) were among the first epigenetic therapies to obtain the United States Food and Drug Administration (US-FDA) approval.

The hypomethylating agents have created a niche for themselves among hematological malignancies, most notably against myelodysplastic syndrome (MDS) [26–30]. However, the application of these hypomethylating agents/DNMT inhibitors (DNMTi) in solid malignancies has met with a snag as the results have not been very encouraging [31, 32]. This could have resulted from the much more complex nature of solid tumors as compared to hematological neoplasms [33]. Another possibility could be due to the slow rate of replication dependent incorporation of the DNMTi agents in the solid tumor cells. Besides, this group of drugs gets inactivated by cytidine deaminase enzyme. Further, the DNMTi agents are effective against solid tumors, at much higher doses than the low doses at which they are effective against hematological malignancies, thus giving rise to the problem of severe toxicity. However, results of a phase 2 trial [34] clearly attest to the fact that low-dose azacytidine can be utilized for chemosensitisation of solid tumors to conventional chemotherapy, thus providing a new direction to the application of DNMTi against solid tumors and paving the way for future clinical studies in this direction.

Another unique strategy to achieve gene demethylation is by using small nonnucleoside DNMT inhibitor molecules (Table 1) that partially competitively inhibit DNMT 1 and decrease the affinity of DNMT for its substrates, thus finally favouring the dissociation of DNMT 1 from hemimethylated DNA. Besides monotherapy, the DNMT inhibitors, for instance, the azanucleosides, have shown excellent efficacy in combination with standard nucleoside analogues like 5-fluorouracil as the DNMT inhibitors can reignite the dormant or silenced proapoptotic genes [35, 36]. Additionally, it has also emerged that HDAC inhibitors and DNMTi when used synergistically may yield superior results, thus potentially opening up another manner of utilization of these agents [37–39].

## 3. Histone Modification and Cancer

Despite DNA methylation being discovered earlier, however, in the recent years, it is the histone modification that has slowly risen to prominence in research in cancer. The remarkable diversity afforded by the versatile histone modifications is slowly being unravelled. The ability of multiple histone modifications to coexist, not all of them being activating in nature as evidenced by the discovery of “bivalent domains,” that is, the coexistence of activating and repressive marks at the promoter sites of developmentally critical genes, provides a dynamic and complex epigenetic landscape, where genetic expression can be modulated at various levels and in different manners. Let us then try to elaborate on the role played by different types of histone modifications in cancer.

### 3.1. Histone Acetylation: An Untapped Gravy Train

Histone acetylation, a very dynamic histone modifying interaction, is carried out by histone lysine acetyltransferases (KATs). There exist 2 different types of KATs, that is, types A and B that carry out histone acetylation of nuclear and cytoplasmic histones, respectively. KATs are among the pioneering enzymes to have been implicated in cancer [40]. Numerous instances implicating KATs in various recurrent chromosomal translocations (e.g., MLL-CBP [41], MOZ-TIF2 [42]) and coding mutations (e.g., p300/CBP [43, 44]) in a wide variety of solid and hematological malignancies have been observed. The research into the role of KAT in the causation of malignancies is still in its nascent stages. However, information regarding the consequences of certain translocations has emerged now. It is believed that the MOZ-TIF2 translocation may lead to a return of aggressive leukemia as seen in animal models. Further, it can confer pluripotency and may lead to the reinitiation of self-replicating ability, if introduced into committed hematopoietic progenitor cells. The oncogenic potential of this translocation has a lot to do with the intrinsic and acquired KAT activity [42, 45]. Despite it being apparent that histone acetylation is disturbed in cancers [46, 47], it is extremely interesting to note that several nonhistone proteins also undergo dynamic acetylation, among which oncogenes and tumor suppressor genes like MYC, p53, and PTEN are also included [48]. This has posed a big conundrum towards obtaining a lucid understanding of the specific molecular mechanisms via which KATs contribute to human malignancies. Further, the endeavour aimed at development of specific inhibitors of KAT families has often been fraught with frustration over the issue of specificity of action [49]. But the recent successes so obtained with various derivatives of natural KAT 1 containing compounds in the form of garcinol and curcumin, to name a few, has raised the hopes of the scientific community that specific KAT inhibitors could possibly be synthesised in the near future [49].

### 3.2. Histone Deacetylation: Lying in Wait for Its Second Coming

Histone deacetylation, induced by HDACs, is another epigenetic target that is being explored aggressively in the field of cancer. HDACs carry out the reversal of acetylation at lysine residues. As noted earlier in this review, there exist 4 different classes of HDACs. Classes I, II, and IV HDACs act in a similar manner that requires the presence of a zinc metal ion at the catalytic site, but not a cofactor. However, class III HDACs, especially Sirtuins 1–7, require NAD^+^ (nicotinamide adenine dinucleotide) as a cofactor for their activity. It has been observed that the level of HDACs varies significantly in various types of cancers, especially in case of hematological malignancies. The various chimeric fusion proteins like PML-RAR*α* and AML1-ETO seen in leukemia have been observed to attract various HDACs with an aim of inducing abnormal gene silencing. This in turn contributes to perpetuation of leukemia [50]. Thus, it was hypothesised that inhibitors of the HDACs could in turn lead to reversal of aberrant gene silencing, which could cascade out to induction of growth arrest, differentiation, and apoptosis of the malignant cells [50, 51]. This paved the way for various studies that were carried out with the sole aim of identifying and examining the efficacy of various agents that could reverse the action of HDACs in various types of cancers [50, 51]. Earliest instance of HDAC inhibition, although weak, was recognized with sodium butyrate which included sodium valproate and phenyl butyrate [52–56]. Since then, a range of more potent and structurally diverse HDACi have been obtained naturally or synthetically produced.

Vorinostat, a pan HDACi (inhibitor of classes I and II HDAC enzymes), was granted US-FDA approval on 6 October, 2006, as an oral agent against cutaneous T-cell lymphoma, which can be progressive and persistent and relapsed after 2 cycles of systemic chemotherapy on the basis of phase 1 [57] and phase 2 clinical trials [58, 59]. It acts by binding to the active site of histone deacetylases and chelating the zinc ions found on the active site, thus preventing the deacetylation from occurring, which via cascade of events promotes cellular differentiation. Vorinostat has exhibited activity in various other hematological [60–64] and solid tumors [65], thus making it a promising drug in the years to come.

Another HDACi agent, romidepsin (depsipeptide, FR901228, FK228, and NSC 630176), a natural product obtained from* Chromobacterium violaceum*, received US-FDA approval as a second-line agent for cutaneous T-cell lymphoma on 5 November, 2009. Romidepsin, a prodrug upon entering the cell, is converted into an active compound that preferentially interacts with the zinc at the active site of the HDAC class I enzymes. But it does exert certain amount of inhibitory activity over class II HDAC enzymes [66]. Results from multiple phase 1 trials [67, 68] and subsequently two phase 2 [69, 70] trials with romidepsin showed good response rates. These trials revealed romidepsin to possess a very long duration of action, extending beyond 3 years in some patients, even after discontinuation of the drug, and belied the fear of possessing pronounced cardiac side-effects [71].

In the same class of drugs, MGCD0103, an isotype specific aminophenylbenzamide that inhibits HDAC I and IV enzymes, was being investigated in clinical trials for its utility in various hematological tumors [72, 73] and lymphomas [74]. However, recent reports of pericarditis and pericardial effusion have thrown a spanner in the development process of this drug. Panobinostat (LBH-589) is being developed, with the primary focus being against hematological malignancies [75–77], refractory or relapsed T-cell lymphomas [78, 79], Hodgkin's lymphomas [80], and Waldenstrom macroglobulinemia [81]. Recently, the results of PANORAMA 1 and 2 trials [82] exhibited that panobinostat if added to bortezomib and dexamethasone could significantly extend the progression-free survival period in refractory or relapsed multiple myeloma when compared to bortezomib plus dexamethasone regimen alone [83]. This in turn led to the US-FDA granting panobinostat “PRIORITY REVIEW” designation as a new drug for multiple myeloma in May, 2014. Belinostat (PXD 101), a novel inhibitor of enzymatic activity of class 1 and class 2, has been approved by the US-FDA on 03 July, 2014, for relapsed or refractory peripheral T-cell lymphoma. However, belinostat has not shown similar efficacy against solid tumors [84, 85]. Other HDACi in various stages of development have been listed in Table 2.

Among other molecules that exert their effects via modulation of histone modifications, class III HDACs, known as Sirtuins or the silent information regulator 2 (sir2) family of proteins, have captivated the interest of scientific community in the last decade. This can be attributed to their critical role in several biological processes, which in turn has sparked off multiple studies aimed at exploring their activity against diseases across the spectrum, with special emphasis on tumors. The most potent sirtuin activator, and the first to be characterized, resveratrol (3,5,4′-trihydroxy-trans-stilbene), is a nonflavonoid polyphenol, first isolated in 1940 [86] as an ingredient from the roots of white hellebore (*Veratrum grandiflorum* O. Loes). Based upon its ability to influence various cell-signaling molecules, resveratrol is being explored for its chemosensitising property [87–91] and ability to prevent cancer [92]. However, similar to KATs, HDACs too suffer from issues related to nonspecificity of their activity. Besides interacting with histone proteins, HDACs further interact with nonhistone substrates like nonchimeric oncogenes BCL6 [93]. Thus, presently, despite the large number of molecules being investigated as HDAC inhibitors, the off-target effects of these agents still manage to confound the researchers and pose the single biggest hurdle towards their emergence as an effective therapeutic option against cancer.

### 3.3. BET (Bromodomain and Extraterminal Domain Family) Inhibitors: The New Kid on the Block

In addition to histone acetylation and deacetylation, another avenue for exploration in the form of histone acetylation readers has slowly emerged that has been shown to exert influence over genetic expression. These readers comprise a binding motif termed as bromodomain that is very well conserved over generations [94]. Of the many groups of proteins belonging to the family of histone acetylation readers, recent studies have illustrated that the BET family containing the bromodomain proteins (BRD2, BRD3, BRD4, and BRDt) can be successfully inhibited [95, 96]. The proteins belonging to the BET family possess tandem amino-terminal bromodomains that are highly conserved sequentially. Targeting the BET family of proteins assumes significance as they are fundamental to transcriptional elongation and cell-cycle progression [96]. BET inhibitors have been found to be very effective against nuclear protein in testis- (NUT-) midline carcinoma [96] and in a wide spectrum of hematological malignancies [97–100]. It has been hypothesised that the antineoplastic activity of BET inhibitors could be attributed to myelocytomatosis (MYC) transcription downregulation. MYC is a master regulatory gene for cellular proliferation and survival and is very commonly dysregulated in neoplastic conditions [101]. However, the results of a few studies have produced evidences that suggest that the effects of BET inhibition may not be solely due to MYC inhibition [97–99]. Amidst all the controversy surrounding BET inhibitors, it has however emerged that BET inhibitors can specifically modulate a small set of genes, primarily by inhibiting transcription elongation. Further studies are needed to decipher the other mechanisms by which BET inhibition can occur and the associated efficacy of each mechanism that can be translated into potential anticancer therapeutic options.

### 3.4. Histone Methylation and Demethylation: The Rising Star of Cancer Epigenetics

Histone methylation can occur at arginine, histidine, or lysine residues on the histone side chains. However, we shall limit our discussion to methylation of lysine residues only as they characterize histone methylation best. The enzyme implicated here, that is, histone lysine methyltransferases (KMT), unlike the KATs possesses the ability to target specific lysine residues, thus enabling the scientists to leapfrog the issue of off-target effects that has often proven to be a big hindrance in the development of epigenetic agents. Various cancers have been studied in relation to a large number of KMTs that include MMSET, EZH2, and MLL family members. However, in recent years, among the KMTs, it is the paradoxical role of EZH2 in human malignancies that has captured the imagination of the epigeneticists [102]. Overexpression of EZH2 which is responsible for H3K27 methylation, in prostate and breast cancer, was found to be associated with poor prognosis [103, 104], suggestive of EZH2 being an oncogene. However, a few studies also showed that coding mutations in EZH2 were present in various lymphoid and myeloid neoplasms, which implied that EZH2 could potentially have a tumor suppressive role [105–108]. The exactness of the mechanisms by which EZH2 impacts cancer needs further investigation. However, in recent times, a better understanding of the role of KMTs in various types of cancers has accelerated the search for specific KMT inhibitors, especially in the light of the promise exhibited by small molecule inhibitors in MLL leukemia [109].

Alternatively, the discovery of two different classes of histone lysine demethylases (LSD) forced the scientists to reconsider the earlier held notion that histone lysine methylation was a stable epigenetic modification. The first class of lysine histone demethylases, that is, LSD1/KDM1A, carries out amine oxidation leading to demethylation of lysine in presence of flavin adenine dinucleotide (FAD) as a cofactor. However, its activity is limited to mono- and dimethyl lysine as it requires a protonated nitrogen atom for its activity. On the other hand, the second class of histone lysine demethylases, that is, Jumonji demethylases, with a well-conserved JmjC domain, functions through an oxidative and radical attack mechanism. As the Jumonji class of enzymes does not require a free protonated nitrogen atom to initiate its activity, the spectrum of substrates on which it can exert its activity also includes trimethyllysine. Further, these enzymes' activity is guided by the multisubunit complexes within which they reside that in turn are responsible for the specificity of their activities. For instance, LSD1 functions as a repressor of transcription when it is associated with the corepressor for RE1-silencing transcription factor (Co-REST) complex. However, it functions as an activator, when it is associated with androgen receptor [110]. Research into the role of histone methylation and demethylation in cancer is still in its infancy. However, the emergence of data from various preclinical studies indicating their therapeutic potential in acute myeloid leukemia (AML) will further spur on the development of histone demethylases inhibitors, which are already at various stages of development [111, 112].

### 3.5. Histone Methylation Readers and Histone Phosphorylation: The Path Less Trodden

Analogous to DNA methylation readers, histone lysine methylation too is recognized and analysed by various proteins that possess special motifs. These specialised domains can be broadly classified as the Royal Family including Tudor domains, Chromo domains, malignant brain tumor domains, and PHD fingers [113]. Recent studies have come up with evidences to implicate a few of the lysine methylation readers in various types of cancers. For instance, the expression of a chromodomain protein HP1 has been observed to be altered [114]. Another example in the form of coding mutations among ING family members that specifically recognizes H3K4me3, being identified in melanomas and breast cancer, points out to the potential of histone methylation readers being developed as antineoplastic agents [115]. Further, a recent study examined AML induced by the fusion of NUP98 and PHD finger containing a segment of JARID1A or PHF23. It was observed that mutations that counter PHD finger's ability to bind to H3K4me3 could indeed suppress the development of this particular variant of AML [116]. Thus, despite the paucity of research in this section of epigenetics, the above instances do exemplify the potential that histone lysine methylation readers possess as antineoplastic agents.

Besides histone methylation, another highly dynamic posttranslational histone modification that is now attracting the attention of epigeneticists is histone phosphorylation. Histone phosphorylation is essential to various cellular phenomena, for instance, mitosis and apoptosis. It is regulated by kinases and phosphatases that carry out phosphorylation and dephosphorylation, respectively. Though very little is known about the role of phosphatases outside the realms of DNA repair and mitosis regulation, kinases on the other hand have been studied at great length. Till recently, kinases' role was thought to be limited to signal transduction in the cytoplasm. However, now, certain kinases are believed to be responsible for histone phosphorylation [117–119], most prominent of which is JAK2 (Janus Kinase 2). JAK2 has been observed to be overexpressed in various hematological malignancies. It has been shown to phosphorylate H3Y41, thus preventing the binding of chromatin repressor HP1*α* and leading to the activation of various hematological oncogenes. Further, the research into JAK enzymes broadened out to include Hodgkin's lymphoma and mediastinal B-cell lymphoma that shared the same mechanism [120]. In light of the fact that various small molecules that are known to inhibit various kinases are already in use as anticancer agents and these agents have been observed to decrease the histone phosphorylation, extensive research into JAK inhibitors was carried out. Ruxolitinib, which selectively inhibits JAK1 and JAK2 enzymes, was the first JAK inhibitor to be approved for treatment of myelofibrosis in 2011 by US-FDA. JAK1 and JAK2 are responsible for recruiting signal transducers and activators of transcription to cytokine receptors leading to modulation of gene expression. Ruxolitinib inhibits the dysregulated JAK signaling associated with myelofibrosis. The phase 3 Controlled Myelofibrosis Study with Oral JAK Inhibitor-1 (COMFORT-1) and COMFORT-2 trials showed significant benefits by reducing spleen size, relieving debilitating symptoms, and improving overall survival. This drug has been hailed as a potentially blockbuster drug. It had gained very rapid acceptance among the US hematologists and oncologists.

### 3.6. Chromatin Remodelers as Anticancer Agents: Fool's Gold or Reality

Chromatin remodelers can be broadly divided into four main classes: the switching defective/sucrose nonfermenting (SWI/SNF) class, the imitation SWI (ISWI) class, nucleosome remodeling and deacetylation (NuRD)/Mi-2/chromodomain helicase DNA binding (CHD) class, and finally inositol requiring 80 (INO80) class. These enzymes carry out relocation, removal, and exchanging of histones by utilising ATP as the energy source. These chromatin-remodeling complexes play a central role in regulating various key biological phenomena, for instance, DNA replication and repair; apoptosis; chromosome segregation; and development and pluripotency.

However, it has now come to light that many of the chromatin-remodeling families may undergo mutation in various types of malignancies [121–123]. This has been further bolstered by the evidence that has emerged after cancer genomes were sequenced out. However, in the backdrop of these new findings, it becomes pertinent to be established beyond reproach that SWI/SNF mutations can lead to an imbalance between self-renewal and maturation, thus favouring a state of malignancy. Further, a lack of mechanistic insights into the process of oncogenesis induced by mutations in chromatin-remodeling complexes has put a spanner in the works and hampered the development of chromatin remodelers as effective anticancer agents.

## 4. Future Challenges

Despite the recent advances in the field of cancer epigenetics, the central maxim of tumorigenesis that it is primarily a process triggered and perpetuated by genetic anomalies remains largely unchallenged. However, notwithstanding the importance of genetic mutations, it has become clear that malignant metamorphosis of cells is governed to a large degree by the cellular environment. The packaging and the manner of packaging of genes hold as much significance as the genome itself in the modulation of cellular functions that are critical to determining cellular identity and also for producing malignant states. It has been proven beyond reproach now that the various tell-tale features of cancer like self-renewal, prevention of differentiation, skirting around cellular death, and apoptosis and tissue invasion are greatly influenced by epigenetic regulatory processes.

Despite these contentions, few questions still linger over the applicability of epigenetics as anticancer agents. Despite the assertion that epigenetic regulators may control only a few selective genes and hence could possibly affect the target genes selectively, mechanistic insights into how the epigenetic regulators target these genes continue to elude the researchers. Further, what are the features of these genes that attract the epigenetic regulators?

Additionally, from the evidences that have emerged, it is clear that epigenetic therapies have found more favour among hematological malignancies. Could this be due to the innately more complex nature of the solid malignancies? Could the intrinsically different cellular nature have anything to do with this? Are we using the epigenetic agents in the correct manner? Should the various epigenetic agents be combined together or are they better as solo therapeutic agents? The underlying biological reasons behind this need to be explored and understood urgently if epigenetic therapies are to stand a chance at finding application against solid malignancies.

In spite of these significant queries, the knowledge of epigenetic regulators has thrown up potential therapeutic marks in plenty. It is not just the various enzymes, but various modification readers, for instance, acetylation readers and methylation readers, have also attracted the attention of the pharmaceutical industry. High throughput drug discovery programs targeting cancer are being carried out [111, 124]. Though the path towards making epigenetic agents, an effective anticancer therapeutic option, is riddled with many setbacks and failures, the scientific data obtained from various epigenetic studies till date forms the bedrock on which the future of epigenetics in cancer can be built upon.

## Figures and Tables

**Table 1:** DNMT inhibitors in cancer.

Drug	Therapeutic use	Developmental stage
*Nucleoside analogue inhibitors*		
(1) 5-azacytidine	Myelodysplastic syndrome	Approved [United States-Food and Drug Administration (US-FDA)]
	Acute myeloid leukemia	Phase 2
	Solid tumors	Phase 2
(2) Decitabine	Myelodysplastic syndrome	Approved (US-FDA)
	Acute myeloid leukemia	Approved [European Commission (EC)]
(3) Zebularine	Solid tumors like breast, urinary bladder, hepatocellular cancer	Preclinical
(4) SGI-110	Myelodysplastic syndrome	Phase 1
	Acute myeloid leukemia	Phase 1
	Solid tumors like bladder cancer	Preclinical

*Nonnucleoside analogue inhibitors*		
(1) Procainamide	Solid tumors like bladder, breast, prostate, cervix	Preclinical
(2) Procaine		
(3) Epigallocatechin-3-gallate		
(4) SGI-1027	Leukemia	Preclinical
(5) Hydralazine	Breast cancer	Phase 2
	Ovarian cancer	Phase 3
	Cervical cancer	Phase 3

**Table 2:** HDAC inhibitors in developmental phases.

Drug	Therapeutic use	Developmental stage
(1) Panobinostat	Cutaneous T-cell lymphoma	Phase 3

(2) Valproic acid	Cervical cancer	Phase 3
	Ovarian cancer	Phase 3

(3) Belinostat	Ovarian cancer	Phase 2

(4) Mocetinostat	Follicular lymphoma	Phase 2
	Hodgkin's lymphoma	Phase 2
	Acute myeloid leukemia	Phase 2

(5) Abexinostat	Sarcoma and lymphoma	Phase 2

(6) Entinostat	Hodgkin's lymphoma	Phase 2
	Breast cancer	Phase 2
	Metastatic lung cancer	Phase 2

(7) SB939	Prostate cancer	Phase 2

(8) Resminostat	Hodgkin's lymphoma	Phase 2
	Hepatocellular carcinoma	Phase 2

(9) Givinostat	Leukemia and lymphoma	Phase 2
	Myelofibrosis	Preclinical

(10) Kevetrin	Ovarian cancer	Phase 1

(11) ACY-1215	Multiple myeloma	Phase 1
